# Early referral of trauma patients to dedicated trauma psychology service: An observational study screening for post-traumatic stress disorder and depression

**DOI:** 10.5339/qmj.2025.17

**Published:** 2025-03-08

**Authors:** Tulika Mehta Agarwal, Jain Varghese, Ayman El-Menyar, Amer Mohammad Abualjoud, Jenalyn Amedina Salvador, Ammar Al-Hassani, Hassan Al-Thani

**Affiliations:** ^1^Department of Surgery, Trauma Surgery, Hamad General Hospital, Doha, Qatar; ^2^Department of Clinical Medicine, Weill Cornell Medical School, Doha, Qatar; ^3^Clinical Research, Trauma & Vascular Surgery, Department of Surgery, Hamad General Hospital, Doha, Qatar *Correspondence: Ayman El-Menyar. Email: aymanco65@yahoo.com

**Keywords:** Screening, psychology, trauma, depression, PTSD, ITSS

## Abstract

**Background:**

The screening of post-traumatic stress disorder (PTSD) and depression after an injury is essential for improving the patient's quality of life. The aim of this study was to assess the utility of PTSD and depression screening and the early referral to the trauma psychology service at a level 1 trauma center. We hypothesized that as the screening process becomes more established as a standard of care, compliance with screening would improve. Furthermore, early referral of trauma patients to a dedicated psychologist within the trauma care system would be beneficial.

**Methods:**

This retrospective study involved 1,245 consecutive eligible patients who were admitted to the trauma service between September 2019 and December 2020. The Injured Trauma Survivor Screen (ITSS) and an additional criteria checklist were used for patient screening, and data were analyzed. The screening was conducted within 24 hours of the admission of trauma patients aged  ≥ 14 years, all of whom had a Glasgow Coma Scale of 15.

**Results:**

The findings of the study showed that the integration of the new screening tool into a standard of care requires a significant amount of time. Screening compliance increased from 84% to 100% throughout the duration of the study. Notably, there was a 10% gap in the referral of patients identified through the ITSS tool, with 64% referrals based on the symptom checklist.

**Conclusions:**

The current screening methods used as a standard of care show good utility value in identifying trauma patients predisposed to developing PTSD or depression, warranting their continued use. Facilitating direct referrals to trauma psychology service by attending staff, including nurses, could help bridge the gap in patient identification and referral. However, further research is warranted to validate this process.

## Introduction

Traumatic injuries are common causes of disability, hospitalization, and psychiatric disorders, making them a serious public health concern.^1^ Despite methodological variations across various studies, there is a general consensus that at least 50% of the population reports trauma exposure.^2^ Individuals who experience traumatic events often face temporary difficulty in their ability to adapt and manage their circumstances. Trauma survivors encounter physical, emotional, and financial consequences that can influence both their personal lives and the well-being of their families. The incidence of post-traumatic stress disorder (PTSD) and depression are common among these individuals.^3^ Therefore, there has been an increasing focus in recent years on understanding the prevalence of PTSD and depression in trauma survivors. Trauma patients can develop PTSD and/or depression at any stage following a traumatic event, necessitating the incorporation of psychological care into trauma management. Research indicates that 10–20% of traumatic injury survivors may develop PTSD,^4^ while the prevalence of major depressive disorder ranges from 9% to 15%.^5^ These figures can be significantly elevated in cases involving acute orthopedic trauma, with reported rates of 32.6% for depression and 26.6% for PTSD.^6^

Depression is a mood disorder that causes a persistent feeling of sadness and loss of interest. The American Psychiatric Association's Diagnostic Statistical Manual of Mental Disorders, Fifth Edition (DSM-5) classifies depressive disorders as disruptive mood dysregulation disorder.^7^

PTSD is classified as a stress-related disorder in the Diagnostic and Statistical Manual (version 5).^8^ This condition is serious and can be potentially debilitating, affecting individuals who have either witnessed or experienced traumatic events. The symptoms associated with PTSD can be grouped into four categories: intrusion symptoms, persistent avoidance, negative alterations in cognitions and mood, and marked alterations in arousal.8

Symptoms of depression commonly occur after a traumatic event, sometimes in conjunction with PTSD.^6^ Therefore, it becomes crucial to integrate psychological aspects of patient care with trauma management. Approximately 84% of individuals suffering from PTSD or major depression may have comorbid conditions such as alcohol or drug abuse, feelings of guilt, and employment problems, all of which make their daily life more difficult. The coexistence of depression and PTSD can lead to a poor response to psychotherapy, highlighting the importance of addressing both conditions. Therefore, early detection and appropriate treatment are crucial. Treatment for PTSD assists the patient in tolerating and assimilating the sense of “traumatic helplessness” that they experience.^9^ A screening conducted for PTSD/depression one month following a traumatic event among 139 participants who were admitted to a level one trauma center showed that 40 individuals met the criteria for PTSD, while 28 were diagnosed with depression.^9^ Many studies have shown the relationship between depression and traumatic injury events. In a study involving 500 trauma survivors with non-neurological injuries, 68.4% of patients screened positive for depression but only 9.8% received treatment.^10^

There is a growing body of literature on trauma and PTSD among the civil population of the Middle East.^11^ However, most trauma centers do not conduct any assessment for predisposition to PTSD/depression. This gap highlights the necessity for assessing patients who have experienced traumatic injuries to determine their risk, enabling referrals to the in-house trauma psychology service for appropriate care. Early detection of such predispositions can facilitate the holistic reintegration of patients with traumatic injuries. The present study addresses the utility of early identification of predisposition to PTSD/depression and the gaps in the referral process. This study used the Injured Trauma Survivor Screen (ITSS) tool along with the additional criteria checklist to identify trauma patients who may be at risk of developing PTSD or depression. The ITSS is one of only two well-validated scales that can be administered within the first 24 hours of a patient's admission, effectively screening for both PTSD and depression. The selection of this scale over the patient adjustment scale was primarily due to its simplicity, as it required patients to respond with only “yes” or “no” to the posed questions.^12^ In contrast, all other available tools can be administered only after 15–30 days from the day of the injury.^12^ The additional criteria checklist was used as a secondary screening measure to ensure that all patients with injuries and circumstances affecting their post-injury rehabilitation were referred to a psychologist, irrespective of their ITSS score severity. This approach would promote a smoother rehabilitation process following discharge.

Furthermore, the early referral of trauma patients to a dedicated psychologist within the trauma care system, coordinated by the treating team including nurses, would be beneficial. It is hypothesized that as the screening process becomes established as a standard of care, compliance with screening will improve.

The objective of this article was to assess the predisposition to PTSD and depression in patients with traumatic injuries, with timely referrals to the trauma psychology service within the trauma surgery department.

## Methods

**Figure 1.**
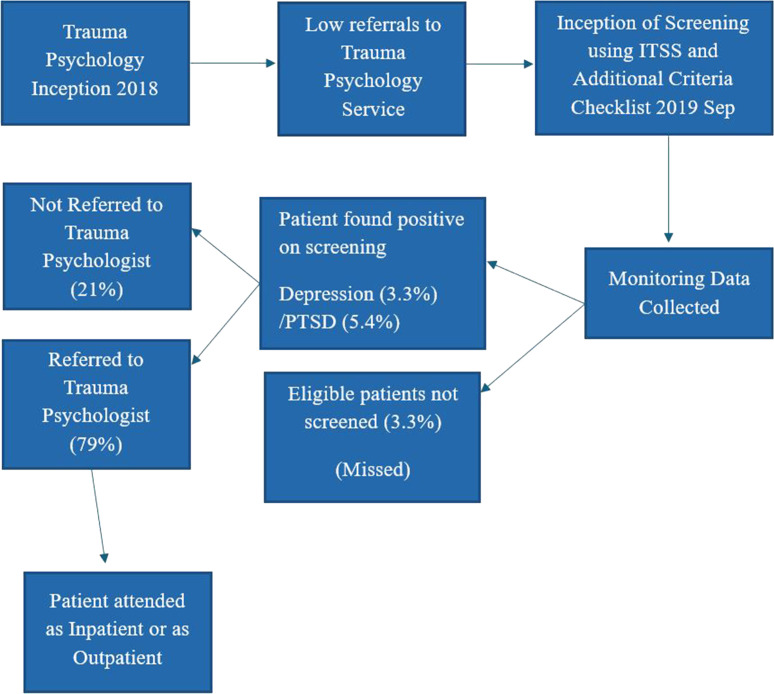
Flowchart of trauma psychology: screened, identified, and referred patients.

This study used a retrospective case study design, which collected, analyzed, and descriptively reported Cerner data related to trauma patients admitted to the Trauma Surgery Unit for 16 months (September 1, 2019 to December 31, 2020). The data of all patients that met the inclusion criteria were included in this study, which used a non-probability purposive sampling method. The expected outcome of this study was to have a positive impact on the psychological care and recovery of trauma patients following their traumatic experiences. Figure 1 shows the trauma psychology service flowchart at the HTC (Hamad Trauma Center) in Hamad General Hospital.

**Figure 2.**
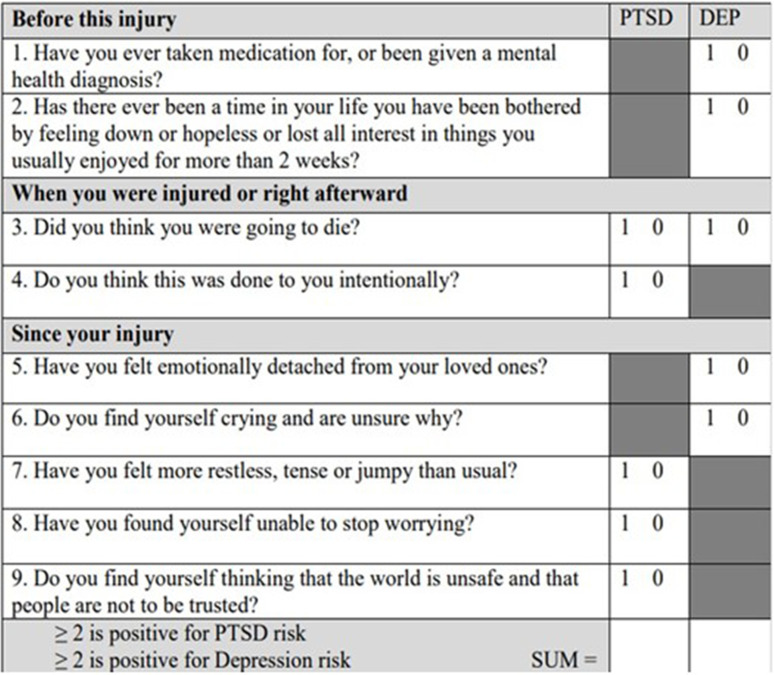
Injured Trauma Survivor Screen (ITSS) adopted from https://cdn-links.lww.com/permalink/ta/a/ta_82_1_2016_11_21_hunt_16-05529_sdc1.pdf. 1 = yes, 2 = no.

Data collection involved screening patients admitted to the Trauma Surgery Unit with the ITSS tool (Figure 2) within the first 24 hours of their admission to the trauma ward. The ITSS is a nine-item questionnaire specifically designed for trauma survivors following an acute traumatic event. This brief and simple instrument effectively captures an accurate description of patient characteristics such as behaviors, thoughts, and views, thereby aiming to reduce the incidence of untreated cases of PTSD and depression.^4^ The ITSS serves as a standardized tool designed for the early identification of the potential risk of developing PTSD and depression to prevent long-term psychological disorders that would impair the quality of life of trauma survivors beyond the effect of injury.^9^ A positive response to any of the screening questions is assigned a value of one point, while a negative response receives a score of zero. If the sum of questions 1, 2, 3, 5, and 6 equals or exceeds 2, the screen indicates a high risk of depression. Similarly, if the sum of questions 3, 4, 7, 8, and 9 is equal to or greater than 2, the screen indicates an increased risk of PTSD.^9^ Based on the results, it is necessary to refer patients with a score of 2 or higher to trauma psychology services. The screening was conducted within 24 hours of the admission of trauma patients aged 14 years and above, provided they are conscious, oriented, and have a Glasgow Coma Scale (GCS) of 15 upon their entry to the Trauma Surgery Unit. Patients with pre-existing psychiatric comorbidity or substance abuse issues affecting GCS were excluded from the study. Patients were admitted to this unit once they were relatively stable and no longer in a critical care environment (Emergency Department/TICU). This study used data collected from these patients. Trauma nurses screened the eligible patients through interviews using the standardized ITSS tool.

The trauma psychologist provided training to the nursing staff on the effective implementation of ITSS. Furthermore, an additional criteria checklist was developed in the trauma psychology section based on a review of the literature on types of injury, injury outcomes, and conditions at the time of injury that may increase the likelihood of psychological challenges (Table 1). Trauma patients who received a zero score on the ITSS but met any of the criteria on the additional checklist were identified as having a high risk of psychological challenges or difficulties in coping.

Patients with moderate to severe traumatic brain injuries who were unable to recognize individuals, locations, times, or situations did not undergo the ITSS, as this assessment had not been validated for such individuals. Patients with a low GCS were screened later after they had regained consciousness and orientation. Additionally, patients under the age of 14 were not included in this study because their admission was outside the scope of the Trauma Surgery Unit's care.

The researchers maintained complete confidentiality of the information obtained through participants’ involvement in this study, committing to neither disclose it to third parties nor use it for purposes beyond those specified in this study. However, information may be provided by the researcher if required by directives from the research team, a court, or a government authority. Furthermore, the researcher ensured that all personnel associated with the healthcare operations of the Trauma Surgery Unit complied with confidentiality and non-disclosure requirements.

The number of eligible trauma patients and those screened were tabulated. In addition, the number of patients identified as being predisposed to developing PTSD or depression, based on the ITTS and additional criteria checklist, was compared to those referred to the trauma psychology service of the Trauma Surgery Unit through various modes of communication, such as phone calls, emails, Cerner consultations, or referral orders. Data are presented as number, proportion, and median with interquartile range (IQR) as appropriate.

## Results

During the study period, a total of 1,352 consecutive patients admitted to the Trauma Surgery Unit were assessed for eligibility, resulting in 1,245 patients who met the data collection criteria being enrolled in the analysis. The median age of the patients was 34 years (IQR 26–44 years), with 90.7% being male. The median length of stay in the intensive care unit and the hospital was two days (IQR 2–4 days) and four days (IQR 2–9 days), respectively. A total of 107 patients were excluded from the ITSS due to a low GCS, while an additional 41 patients (3.3%) were not screened as the tool was not administered for various reasons, such as the attending nurse's failure to use the screening tool within 24 hours of the patient's admission to the trauma ward. Figure 1 summarizes the steps involved in the referral process to psychology services.

ITSS tool was used to screen 1,204 eligible patients (96.7%). Initially, when implementing the ITSS tool for screening depression and PTSD in the department, many patients were missing for the screening process. However, this issue gradually improved and stabilized over time.

**Figure 3.**
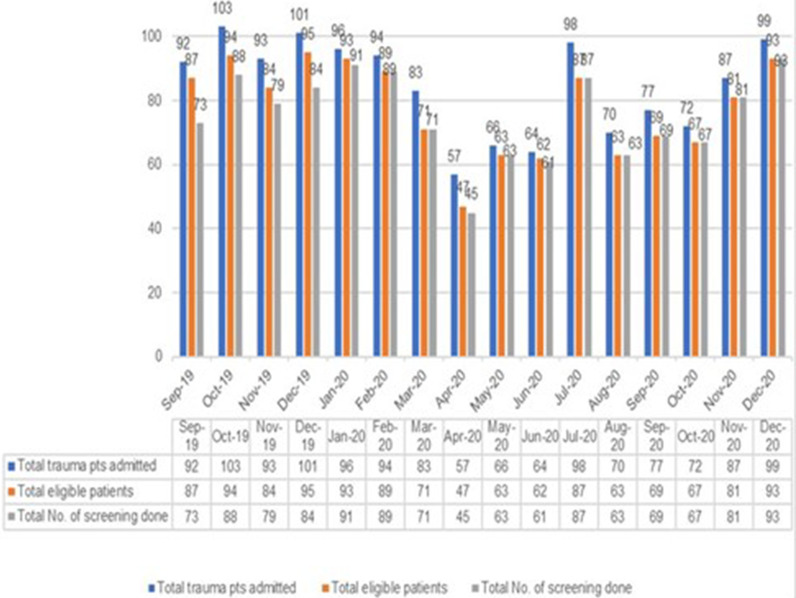
Number of patients admitted, eligible, and assessed during the study period.

**Figure 4.**
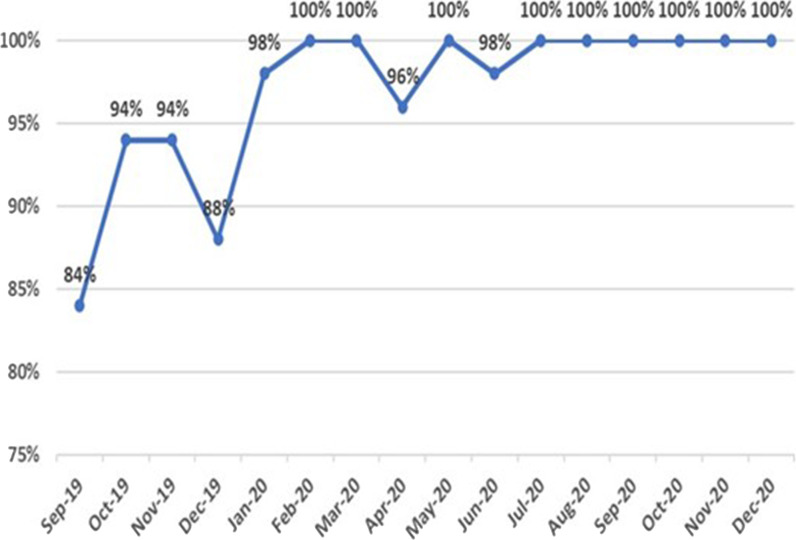
Percentage of eligible patients screened for compliance within 24 hours of admission.

Figure 3 shows the details of the number of patients admitted, deemed eligible, and assessed during the study period. The highest patient admission count was in October 2019 (103 patients), while the lowest was recorded in April 2020 (57 patients). A total of 93 patients were screened in December 2020, making it as the month with the highest number of completed screenings during the study period (Figure 3). The percentage of eligible trauma patients who underwent the ITSS was lowest in September 2019 (84%) and December 2019 (88%). Following these months, there was a notable improvement in compliance with the ITSS, which remained relatively stable for the remainder of the study period (Figure 4).

All screenings were conducted within 24 hours of the patients’ admission to the Trauma Surgery Unit. Using a cut-off score of 2, the ITSS tool was applied to a total of 1,204 patients. Among these, 41 patients (3.3%) were identified as being predisposed to developing depression, while 66 patients (5.48%) were identified as at risk for developing PTSD over the entire study period (Figure 1). Additionally, 27 patients (2.2%) showed a high risk for developing both depression and PTSD. Notably, the predisposition to psychological challenges was higher during the period from April 2020 to May 2020, despite the lower number of screenings conducted during this period in comparison to other months within the study time frame (Figure 3).

**Figure 5.**
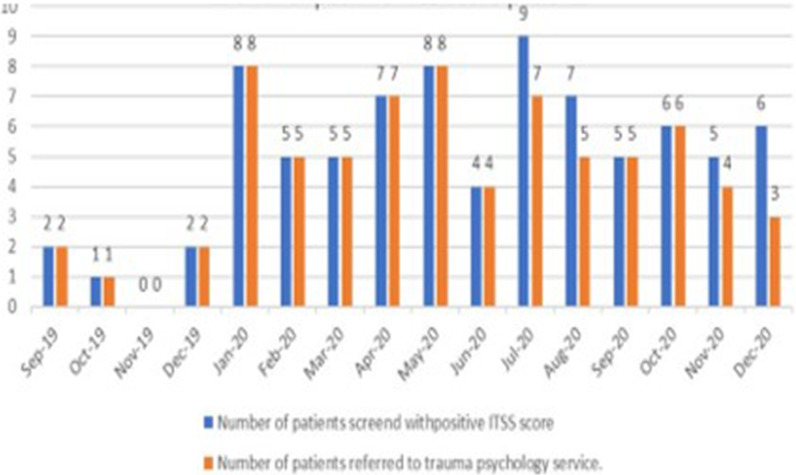
Number of cases identified based on the ITSS scores and those referred to the trauma psychology service.

A 10% gap was evident between the number of identified and referred patients for PTSD or depression. A total of 80 patients were screened as positively predisposed to developing depression or PTSD, of which 72 were referred to the trauma psychology service. Notably, eight patients who responded positively to the risk of developing depression or PTSD on the ITSS tool were not referred to the trauma psychology clinic (Figure 5).

**Figure 6.**
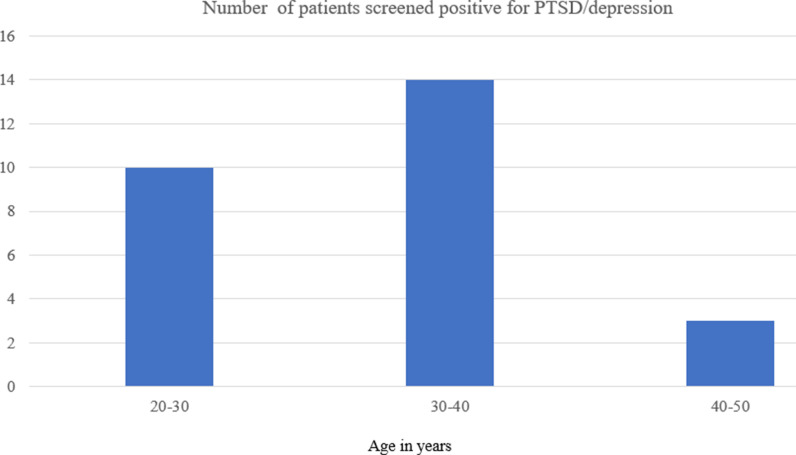
Age distribution of patients screened positive for PTSD or depression.

**Figure 7.**
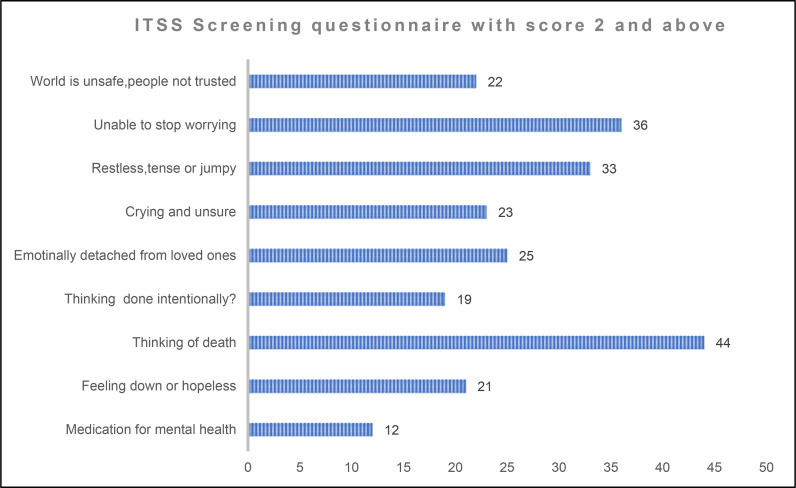
The distribution of responses for each question among positively screened patients.

Figure 6 shows that patients aged between 30 and 40 years (52%) had a higher risk for developing both PTSD and depression. Among the questions from the nine-item ITSS tool, the question “Did you think you were going to die?” received the highest positive responses (44 patients). Additionally, 36 patients responded in affirmative to the question “Have you found yourself unable to stop worrying?” while 33 reported feeling restless, tense, or jumpy. Conversely, the question “Have you taken medication for or been given a mental health diagnosis?” had the lowest positive responses, with only 12 patients responding affirmatively (Figure 7).

Table 1 outlines the additional criteria based on the type of injury, the circumstances of injury, and the resulting impact, which were used in the Trauma Surgery Unit to identify and refer patients to the trauma psychology service. These criteria were applied in conjunction with a positive ITSS score to facilitate trauma psychology referrals. During the study period, 404 patients met one or more of these additional referral criteria. However, only 320 patients were ultimately referred to the trauma psychology clinic, resulting in a referral gap of 20.8%.

Overall, the referrals made to the trauma psychology service, based on the ITSS tool and the additional criteria, accounted for 79% of cases. The highest number of referred patients (91) reported experiencing intolerable pain, which was assessed as a standard of care using the numerical rating scale. Among these patients, 95% were referred for psychological support. Similarly, referrals were notably high for paraplegic patients (89%) and those who had experienced the death of a co-passenger (90%). Conversely, referrals were lowest among patients with grinder injuries (36%) and those with blast-related injuries (47%).

Among the patients who screened positive for either PTSD or depression on the ITSS tool, 22 patients had engaged in self-harm through suicide attempts. A total of 24 patients were admitted following a motor vehicle crash (MVC), 13 patients with a history of falling from a height, 6 patients with assault-related injuries, 5 patients suffered injuries due to the fall of a heavy object, 4 patients with blast injuries and paraplegia, 3 patients had all-terrain vehicle (ATV) injuries, 2 cases for pedestrian injuries, and 1 case each of gunshot wounds, injury from a sharp object, and being kicked by a camel. Suicidal attempt patients had the highest number of positive screenings for both depression and PTSD (Table 2). Patients who had attempted suicide showed a higher predisposition for both PTSD and depression. Furthermore, patients involved in MVC had higher chances of developing PTSD rather than depression, while those who fell from heights showed higher chances of developing depression compared to PTSD. A similar trend was observed for patients who experienced the fall of a heavy object on their body. Additionally, gaps were also identified in the referrals made to the trauma psychology service based on the additional criteria checklist (Table 1).

## Discussion

Psychological challenges, such as depression and post-traumatic distress, are common outcomes following traumatic injuries. Psychological factors significantly influence the recovery process of individuals.^13^ Therefore, it is crucial to diagnose, refer, and treat these disorders as part of the care provided to trauma patients. Patients who are predisposed to developing mental health challenges and show subthreshold symptoms often develop into major depressive episodes, highlighting the importance of addressing these concerns.

According to the guidelines established by the World Health Organization and the American College of Surgeons Committee on Trauma (ACS-CoT) for initiating screening programs,^14,15^ the benefits of implementing such screening measures were thoroughly evaluated before the deployment of the screening tool. The findings indicated that the screening tool effectively identified trauma patients predisposed to developing PTSD or depression in the future. This process also created a pathway for referrals of identified patients to trauma psychology services.

The ITSS serves as a screening tool used in the Trauma Ward of Hamad General Hospital to assess predisposition to develop depression or PTSD. A recent systematic review identified this scale as one of the best scales for assessing predisposition to PTSD. When a patient scores positively on the screening tool or the symptom checklist, they are subsequently referred to the trauma psychology service for further intervention.

During the study period, data collected from the ITSS tool were compiled and assessed to understand the pattern of data collection, response rates, and referrals to the trauma psychology service. The findings showed a low consistency in screening at the program's inception. However, this consistency improved significantly over time, achieving a steady rate of 100% in the final eight months of the study period. A previous study highlighted that factors such as professional roles and identities, beliefs about consequences, and preconditions were important themes for the successful implementation of the screening process.^16^ It was observed that once the screening was established as a standard of care, nurses gained more clarity about their roles in conducting this screening within 24 hours of patient admission. Periodic training sessions effectively addressed emerging questions and enhanced comprehension regarding the importance of screening for trauma patients. Furthermore, improved monitoring of these administrations by senior nursing management and education coordinators might have contributed to this improvement in screening compliance.

This tool successfully identified 3.4% and 5.5% of patients with traumatic injuries as being at high risk for depression and PTSD, respectively, when assessed within 24 hours of their admission to the trauma ward. This rate was much lower than the incidence rate of PTSD (13%) found in a prospective study conducted in Qatar involving patients with traumatic injuries.^10^ One of the possible reasons for this observation could be the exclusion of patients from other units of the Trauma Surgery Unit during the evaluation of predisposition. This situation could also highlight a gap in the administration of the tool or the patients’ inability to effectively respond to the screening tool during the first shift within 24 hours of their admission to the trauma ward. Given that this screening tool is well validated,^17,18^ it is unlikely that the low numbers are a reflection of the tool's validity.

An important finding was that a significant proportion of the patients identified as predisposed to developing depression in the future were also at risk for developing PTSD, indicating a comorbid relationship. The high prevalence of comorbidity between PTSD and major depressive disorder has been established in several studies.^19,20,21^ Furthermore, the data indicated that patients with traumatic injuries are more prone to developing PTSD than depression (Figure 1), which concurs with the results of several other studies.^22,23^ However, research has revealed an interesting finding that individuals who experience violent injuries exhibit a greater predisposition to develop depression than PTSD, both at baseline and an eight-month period following the injury.^24^ The ITSS scores from the study population supported these findings, showing that patients who had been assaulted were more likely to develop PTSD in the future. (Table 2)

Furthermore, the additional criteria checklist highlighted gaps in the referrals made to the trauma psychology service. The observed gaps in the referrals of trauma patients to mental health services could stem from various barriers faced by clinicians, including lack of time, patient resistance, challenges related to staff training, and inadequate referral options.^25^ The importance of clinician referrals to mental health services has also been emphasized in other studies.^26^ It was identified that this dependence on clinicians for making the referrals might have contributed to the existing gaps.

The highest number of patients fell in the age group of 30–40 years (Figure 6), and showed a predisposition to develop PTSD or depression in the future. This trend could be attributed to the demographic characteristics of the population during this period.^27^ However, it is essential to investigate whether additional factors may be contributing to this phenomenon.

A comprehensive analysis of the responses from patients exhibiting a positive predisposition on the ITSS showed that more than 50% of these patients expressed thoughts regarding the possibility of having died during the traumatic event (Figure 7). Addressing topics related to death may serve as a therapeutic implication for dealing with this challenge, as unresolved concerns could potentially lead to the onset of both PTSD and depression.^28^ Furthermore, 45% of patients reported that they were unable to stop worrying. Anxiety has been shown to hinder the healing of injuries. A meta-analysis that included 17 studies found a correlation between stress and wound healing, with an estimated correlation coefficient of *r* = -0.42 (95% CI: -0.51 to -0.32), concluding that stress adversely affects the healing of wounds.^29^

An additional criteria checklist was further used to identify patients who could be predisposed to developing mental health challenges. Chronic pain, often resulting from traumatic injuries, is associated with positive screenings for PTSD and should not be ignored.^30^ Therefore, these findings support the belief that early identification and intervention for psychological challenges can facilitate healing in patients with traumatic injuries.

The inclusion of self-reported pain as an important factor for referral on the checklist is warranted, as it has been identified as the most frequently reported reason for referrals to a trauma psychology service.

Similarly, self-harming behaviors could arise from the interactive effect of major depressive disorder or non-suicidal self-injurious behavior, which associate with the risk of suicide.^31^ Furthermore, suicidal attempts could serve as an indicator of an underlying predisposition to mood disorders or the presence of a fully developed depressive disorder. Therefore, any attempt at self-harm was identified as an important factor for referral in the checklist. It was observed that among the 31 patients who presented with self-harming behaviors, only 26 were referred to the trauma psychology service, indicating a referral gap of 14%.

Injuries such as crush injuries, grinder injuries, or injury outcomes such as amputations or facial injuries may lead not only to physical disabilities but also to alterations in body image, which may adversely affect an individual's self-confidence and their long-term coping mechanisms.^32^

An interesting study found that the impact of amputation on body image is considerably more profound than that on self-esteem, especially in cases involving lower limb amputations.^33^ Additionally, crush injuries may cause psychological trauma.^34^ The gaps in referrals identified through this checklist were highest for grinder injuries (64%) and blast-related injuries (53%). This disparity may stem from poor GCS due to severe injuries or deficiencies in the referral system itself. It is believed that this gap can be addressed by reinforcing and educating nursing staff on the criteria included in the additional criteria checklist.

Furthermore, among the 81 patients identified as psychologically predisposed, a higher number of patients showed a predisposition to develop PTSD rather than depression, irrespective of the injury type. Our findings are in line with results from a systematic review indicating that many people develop PTSD after both intentional and unintentional trauma. Therefore, understanding these trajectories is crucial for effective treatment and health planning.^22^ Early identification of predisposition to mental health challenges can significantly reduce both the overall direct and indirect costs associated with these conditions, ultimately leading to improved outcomes for patients with traumatic injuries.^35,36^

Once the screening process is established as a standard of care, compliance with screening will improve. Notably, during the latter months of 2020, there was consistently no gap between the number of eligible admitted patients and those who underwent screening, indicating 100% compliance.

## Limitations

Retrospective analysis has inherent limitations, including selection bias and missing data. The main limitation of this study is that there was no secondary assessment to determine whether the ITSS tool effectively identified all predisposed individuals or overlooked some. However, this limitation was partly negated by using the trauma symptom checklist, which identified patients based on the type and circumstance of their injuries and was developed for the trauma department based on a literature review. Further validation and multicenter collaboration are required to contextualize these results in relation to previous studies and the underlying hypotheses. The findings and their implications warrant discussion within an extensive framework. Additionally, potential avenues for future research should be highlighted. Although the eustress–distress–PTSD continuum and stress-coping strategies are important elements, they fall outside the scope of this study.^37^. Early identification of PTSD in the context of trauma can be challenging. However, we emphasize that early referral is crucial, with the patient's ability and convenience being the key for effective screening. Our trauma system is supported by a dedicated psychology team and a follow-up clinic, ensuring that the team is well educated in using the tools. As a level 1 trauma center with international accreditation and affiliation with the TQIP-ACS (Trauma Quality Improvement Program-American College of Surgery), we strive to maintain high compliance standards, although sustaining this over the long term presents challenges that require continuous support. We are committed to upholding a high compliance rate, which will be presented in a future study.

## Conclusions

In patients with post-traumatic injuries, the implementation of a new screening tool may require a significant period before it becomes a standard of care, and 100% screening can be achieved. Current screening methods, which are already established as a standard of care, show good utility value in identifying trauma patients predisposed to developing PTSD or depression, and their continued use is recommended. It is essential to further investigate and address the challenges that lead to gaps in the referral of identified patients to the trauma psychology service. Facilitating direct referrals to the trauma psychology service by nursing staff could potentially bridge the gap in both identification and referral processes. However, additional research is warranted to strengthen this approach.

### Ethics statement

This study was approved by the Institutional Review Board**/**Ethics Committee at Hamad Medical Corporation (approval no.: 01-21-331; approval date: 4/7/2021). The authors followed the guidelines set forth by the Strengthening the Reporting of Observational Studies in Epidemiology (STROBE) Statement.

### Acknowledgments

The authors express their gratitude for the administrative and technical support provided by Mr. Evinston Wilson Shalom. The authors would also like to acknowledge the support of Sheeba Mathews, the Head Nurse Trauma Surgery Unit.

### Competing interests

The authors have no conflicts of interest to declare.

### Authors' contribution

**TA** and **AE:** Conceptualization, methods, and data interpretation. **JV**, **MA**, **JS**, and **TA:** Data collection. **TA:** Manuscript draft. **AE**, **AA**, and **HA:** Manuscript review and editing. All authors have read and agreed to the final version of the manuscript.

## Figures and Tables

**Table 1.** Percentage of referrals based on the additional criteria checklist.

Additional criteria	Total cases	Referred cases	Percentage of referred cases

Assault	83	65	78%

Blast injury	32	15	47%

Attempted self-harm	31	26	84%

Crush injury	23	15	65%

Grinder injury	14	5	36%

Intoxication	48	38	79%

Death of a co-passenger	10	9	90%

Facial injuries	24	20	83%

Amputation	12	10	83%

Partial loss of sensation	15	13	87%

Paraplegia	9	8	89%

Quadriplegia	1	0	0%

Loss of vision	6	5	83%

Pain	96	91	95%

Total	404	320	79%


**Table 2.** Number of patients positively screened for predisposition to PTSD and depression based on the type of injury.

Type of injury	Number of patients screened positive	Patients screened positive for depression	Patients screened positive for PTSD	Patients positive for both depression and PTSD

Motor vehicle crash	24	4	18	6

ATV injury	3	2	2	1

Blast injury	4	1	3	0

Camel kick	1	1	1	1

Suicidal attempt	22	19	17	12

Falling from a height	13	2	11	0

Assault	6	2	5	1

Hit by a sharp object	1	0	1	0

Pedestrian injury	2	2	2	2

Fall of a heavy object	5	1	4	2

Gunshot wounds	1	0	1	0

